# GABAergic/glutamatergic imbalance relative to excessive neuroinflammation in autism spectrum disorders

**DOI:** 10.1186/s12974-014-0189-0

**Published:** 2014-11-19

**Authors:** Afaf El-Ansary, Laila Al-Ayadhi

**Affiliations:** Biochemistry Department, Science College, King Saud University, PO box 22452, 11495 Riyadh, Saudi Arabia; Autism Research and Treatment Center, Riyadh, Saudi Arabia; Shaik Al-Amodi Autism Research Chair, King Saud University, Riyadh, Saudi Arabia; Department of Physiology, Faculty of Medicine, King Saud University, Riyadh, Saudi Arabia; Medicinal Chemistry Department, National Research Center, Dokki, Cairo, Egypt

**Keywords:** Autism, Glutamate excitotoxicity, Gamma aminobutyric acid (GABA), Glutamate/GABA, Tumor necrosis factor-α, Interleukin-6, Interferon-gamma, Interferon-gamma-inducible protein 16

## Abstract

**Background:**

Autism spectrum disorder (ASD) is characterized by three core behavioral domains: social deficits, impaired communication, and repetitive behaviors. Glutamatergic/GABAergic imbalance has been found in various preclinical models of ASD. Additionally, autoimmunity immune dysfunction, and neuroinflammation are also considered as etiological mechanisms of this disorder. This study aimed to elucidate the relationship between glutamatergic/ GABAergic imbalance and neuroinflammation as two recently-discovered autism-related etiological mechanisms.

**Methods:**

Twenty autistic patients aged 3 to 15 years and 19 age- and gender-matched healthy controls were included in this study. The plasma levels of glutamate, GABA and glutamate/GABA ratio as markers of excitotoxicity together with TNF-α, IL-6, IFN-γ and IFI16 as markers of neuroinflammation were determined in both groups.

**Results:**

Autistic patients exhibited glutamate excitotoxicity based on a much higher glutamate concentration in the autistic patients than in the control subjects. Unexpectedly higher GABA and lower glutamate/GABA levels were recorded in autistic patients compared to control subjects. TNF-α and IL-6 were significantly lower, whereas IFN-γ and IFI16 were remarkably higher in the autistic patients than in the control subjects.

**Conclusion:**

Multiple regression analysis revealed associations between reduced GABA level, neuroinflammation and glutamate excitotoxicity. This study indicates that autism is a developmental synaptic disorder showing imbalance in GABAergic and glutamatergic synapses as a consequence of neuroinflammation.

## Introduction

The brain’s immune system is controlled by the microglia, a set of cells that when activated can secrete numerous cytokines, chemokines, eicosanoids, proteases, complement and excitotoxins [1]. It is well known that microglial immune cytokines can be activated by, heavy metals (for example, mercury), amyloid, bacteria, and glutamate. During neurodevelopment, the immune cytokines can act as neurotrophic substances, protecting and promoting neurite growth. With excess activation and when chronically activated, these cytokines can be very destructive.

An autotoxic, non-specific immune destruction of neurons, neurites and synaptic connections has been described by McGeer *et al*. [2]. In this process, either systemic immune factors (cytokines) or local immune factors, such as beta-amyloidcan activate the brain’s immune system via activation of astrocytes and microglia. In both instances brain levels of cytokines, reactive oxygen and nitrogen species, cellular immune components, excitotoxins and arachidonic acid are elevated leading to brain dysfunction.

The difference in severity between patients with autism appears to vary with the stage at which the immune/excitotoxic stress arises and its intensity. In humans, it is well known that a considerable amount of postnatal brain development occurs, with the highest synaptogenesis occurring during the last trimester and the first two postnatal years. An excess of extraneuronal glutamate can interfere with neuronal migration patterns, differentiation and synaptic development, resulting in varying degrees of abnormal brain architecture and hence differing severities of autistic features.

Glutamate exerts its effect through metabotropic (mGlu) and ionotropic (iGlu) glutamate receptors localized in the cellular membranes of neurons and glia as neuron-supporting cells. According to their differential affinity for different agonists, ionotropic receptors include N-methyl-D-aspartate (NMDA), kainate (KA), and amino-3-hydroxy-5-methyl-4-isoxazole propionic acid (AMPA) [3]. The metabotropic receptors belong to the G-protein-coupled receptor, and can be divided into group I (mGluR1 and mGluR5), group II (mGluR2 and mGluR3) and group III (mGluR4 and mGluR6-8) according to their primary sequence and pharmacological agonists [4]. The increased probability of epilepsy in patients with autism suggests enhanced glutamatergic signaling with positive correlation between plasma levels of glutamate and the severity of autism and increased expression of mRNAs encoding the AMPA 1 receptor in the cerebellum of autistic patients [5,6].

While in the adult brain gamma aminobutyric acid (GABA) acts as an inhibitory neurotransmitter, during the perinatal period it depolarizes targeted cells and triggers calcium influx. GABA-mediated calcium signaling regulates a number of important developmental processes which include, cell proliferation, differentiation, synapse maturation, and cell death [7]. A dysfunction of the GABAergic signaling early in development leads to a severe excitatory/inhibitory (E/I) imbalance in neuronal circuits, a condition that may account for some of the behavioral deficits observed in patients with autism [8]. The GABA level, glutamate/GABA and glutamine/glutamate ratios are significantly lower in patients with autism compared to normal controls, thus suggesting a possible abnormality in the regulation between GABA and glutamate that might lead to excitotoxicity [9,10].

The overall aim of this study is to confirm that GABA and glutamate synapses are abnormal in autistic patients and to determine how these abnormalities may be associated with one another and with neuroinflammatory responses as a pathway involved in the etiology of autism.

## Material and methods

### Participants and methods

The study protocol followed the ethical guidelines of the most recent Declaration of Helsinki (Edinburgh, 2000). All subjects enrolled in the study (20 autistic male patients and 19 age- and gender-matched controls) had written informed consent provided by their parents and assented to participate if developmentally able. They were enrolled through the ART Center (Autism Research & Treatment Center) clinic (Riyadh, Saudi Arabia). The ART Center clinic sample population consisted of children diagnosed on the ASD. The diagnosis of ASD was confirmed in all subjects using the Autism Diagnostic Interview-Revised (ADI-R) and the Autism Diagnostic Observation Schedule (ADOS) and 3DI (Developmental, Dimensional Diagnostic Interview). The ages of all autistic children who participated were between 4 and 12 years old. All were simplex cases. All were negative for fragile X gene study. The control group recruited from Well-baby Clinic at King Khaled University hospital with mean age of 4 to 11 years. Subjects were excluded from the investigation if they had organic aciduria, dysmorphic features, or diagnosis of fragile X or other serious neurological (for example, seizures), psychiatric (for example, bipolar disorder) or known medical conditions. All participants were screened via parental interview for current and past physical illness. Children with known endocrine, cardiovascular, pulmonary, liver, kidney or other medical diseases were excluded from the study. None of the recruited autistic patients were on special diets or taking alternative treatments.

### Ethics approval and consent

A written consent was obtained from the parents of each individual case, according to the guidelines of the ethical committee of King Khalid Hospital, King Saud University.

### Blood samples

After an overnight fast, 10 ml blood samples were collected from both groups in test tubes containing sodium heparin as anticoagulant. Tubes were centrifuged at 3,500 rpm at room temperature for 15 minutes; plasma was obtained and deep frozen (at −80°C) until analysis.

### Determination of GABA

The quantitative determination of GABA in human plasma was measured using an ELISA diagnostic kit, a product of Immunodiagnostic AG, Dusseldorf, Germany. This assay was based on the method of competitive ELISAs. The sample preparation included the addition of a derivatization reagent for GABA. Afterwards, the treated samples were incubated in wells of a microtiter plate coated with a polyclonal antibody against the GABA derivative, together with an assay reagent containing a GABA-derivative (tracer). During the incubation period, the target GABA in the sample competed with the tracer for the binding of the polyclonal antibodies on the wall of the microtiter wells. GABA in the sample displaced the tracer from binding to the antibodies. Therefore, the concentration of antibody-bound tracer was inversely proportional to the GABA concentration in the sample. A dose-response curve of absorbance unit (optical density at 450 nm) versus concentration was generated using the values obtained from the standards. GABA present in the patient samples (EDTA plasma) was determined directly from this curve.

### Measurement of glutamate

Glutamate level was assessed using an HPLC method [11]. Plasma samples (0.1 ml) were mixed with 5 μl of mercaptoethanol and allowed to stand for 5 minutes at room temperature, then precipitated with ice-cold methanol while being vortexed. Tubes were allowed to stand for 15 minutes in an ice bucket before samples were separated by centrifugation (5,000 rpm for 15 minutes) and the supernatant was collected. The efficiency of the protein precipitation step was assessed by Bradford’s dye-binding method [12]. The protein-free supernatants were processed immediately for HPLC analysis of glutamate.

### Assay of TNF-α

TNF-α was measured using a mouse TNF-α ELISA kit (Hycult Biotech, Uden, Netherlands. The antibody reacts with the natural TNF-α of the rats and identifies membrane- as well as receptor-bound TNF-α. The TNF-α trimer interacts with either of the two types of TNF-R, leading to receptor cross-linking. One unit of Hycult Biotech Mouse TNF-α approximates the bioactivity of 16 units of human TNF-α according to the standard L929 cytotoxicity assays for TNF-α prepared by the World Health Organization (WHO).

### Assay of IL-6

IL-6 was assayed using a Quantikine ELISA kit (R&D Systems, Minneapolis, MN, USA). A microplate was precoated with a monoclonal antibody specific for rat IL-6. Fifty microliters of each standard, control, or sample were placed in separate wells. The reagent was mixed by gently tapping the plate frame for 1 minute after being covered with the adhesive strip provided. The plate was incubated for 2 hours at room temperature and any rat IL-6 present was bound by the immobilized antibody. After washing away unbound substances, an enzyme-linked polyclonal antibody specific for rat IL-6 was added to the wells. Following a subsequent wash step to remove unbound antibody-enzyme reagents, 100 μl of substrate solution was added to each well and the plate was incubated for 30 minutes at room temperature. The enzyme reaction yielded a blue product that turned yellow when the stop solution was added. The intensity measured for the color was in proportion to the amount of rat IL-6 bound in the initial step.

### Assay of IFN-γ

IFN-γ was measured using an ELISA kit, a product of Thermo Scientific (Rockford, IL, USA) [13] according to the manufacturer’s instructions. This assay employs a quantitative sandwich enzyme immunoassay technique that measures IFN-γ in less than 5 hours. The minimum level of rat IFN-γ detected by this product was less than 2 pg/ml read off the standard curve.

### Determination of interferon-γ-inducible protein 16 (IFI16)

The human IFI16 ELISA kit was provided by Bio-Source2 Kallang Avenue, 06-32, Singapore 339407. This immunoassay kit allows for the *in vitro* quantitative determination of human IFI16 concentrations in plasma. The IFI16 ELISA kit applied the quantitative sandwich enzyme immunoassay technique. The microtiter plate had been pre-coated with a monoclonal antibody specific for IFI16. Standards or samples were then added to the microtiter plate wells and IFI16, if present, bound to the antibody precoated wells. In order to quantitatively determine the amount of IFI16 present in the sample, a standardized preparation of horseradish peroxidase (HRP)-conjugated polyclonal antibody, specific for IFI16, was added to each well to ‘sandwich’ the IFI16 immobilized on the plate. The microtiter plate then underwent incubation, and the wells were subsequently thoroughly washed to remove all unbound components. Next, substrate solutions were added to each well. The enzyme HRP and substrate were allowed to react over a short incubation period. Only those wells that contained IFI16 and HRP-conjugated antibody exhibited a change in color. The enzyme-substrate reaction was terminated by the addition of a sulfuric acid solution and the color change was measured spectrophotometrically at a wavelength of 450 nm. A standard curve was plotted relating the intensity of the color (optical density) to the concentration of standards. The IFI16 concentration in each sample was interpolated from this standard curve.

## Results

**Figure 1:**
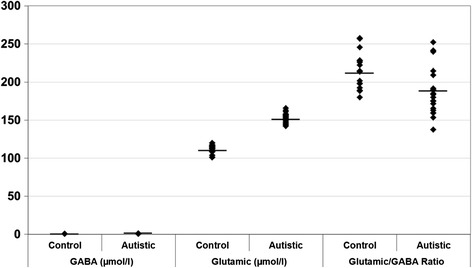
**Gamma aminobutyric acid (GABA) (μmol/l), glutamate (μmol/l) and glutamate/GABA ratio in control and autistic patients.** The mean value for each group is designated by a line.

**Figure 2:**
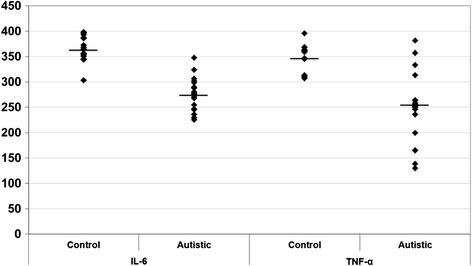
**IL-6 and TNF-α in control and autistic patients.** The mean value for each group is designated by a line.

**Figure 3:**
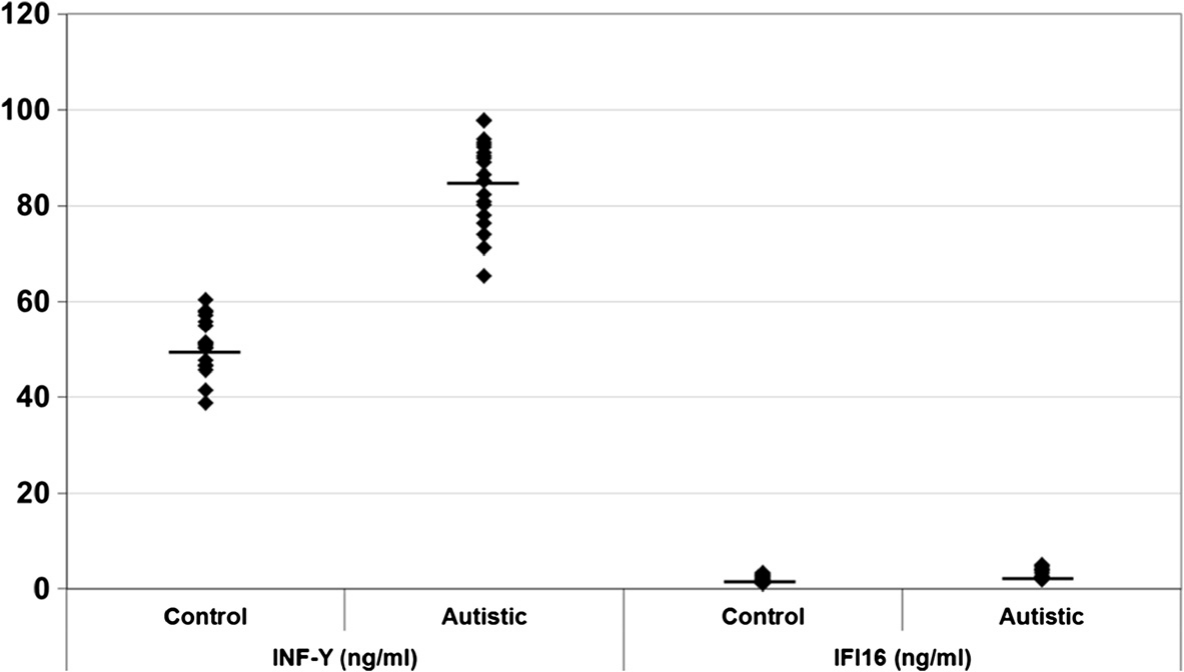
**INF-γ (ng/ml) and IFI16 (ng/ml) in control and autistic patients.** The mean value for each group is designated by a line.

Levels of glutamate, GABA, glutamate/GABA, TNF-α, IL-6, IFN-γ and IFI16 were compared between patients with autism and age-matched control subjects. Data are presented as a mean ± SD of a number of 20 patients with ASD compared to 19 controls, and the significant difference between both groups was presented in Table 1. It was noticed that the six measured parameters differed significantly between patients and controls. Figures 1, 2 and 3 demonstrate individual data distribution around the mean value represented as a straight line for the studied parameters. Table 1 also represents the percentage increase or decrease of the measured parameters of autistic patients relative to control subjects. It can be easily observed that GABA and glutamate recorded 57 and 36% increase respectively in autistic compared to control subjects and about 13% decrease in glutamate/GABA ratio. Table 1 also shows about 25% decrease in TNF-α, IL-6 and around 65 and 62% increase of IFN-γ and IFI16 respectively.

**Table 1:** **Levels of the measured parameters in plasma of autistic patients compared to control**

**Parameter**	**Group**	**N**	**Mean ± SD**	**Percent change**	***P*** **-value**
GABA (μmol/l)	Control	19	0.53 ± 0.05	100.00	0.001
	Autistic	20	0.83 ± 0.12	157.71	
Glutamate (μmol/l)	Control	19	111.91 ± 4.63	100.00	0.001
	Autistic	20	152.80 ± 6.47	136.54	
Glutamate/GABA ratio	Control	19	214.72 ± 22.26	100.00	0.003
	Autistic	20	188.12 ± 30.11	87.61	
IL-6	Control	19	363.49 ± 22.88	100.00	0.001
	Autistic	20	273.87 ± 32.49	75.34	
TNF-α	Control	19	346.23 ± 24.95	100.00	0.001
	Autistic	20	252.35 ± 63.60	72.89	
INF-γ (ng/ml)	Control	19	50.85 ± 5.71	100.00	0.001
	Autistic	20	85.33 ± 9.06	167.80	
IFI16 (ng/ml)	Control	19	1.91 ± 0.60	100.00	0.001
	Autistic	20	3.11 ± 1.01	162.49	

Table 1 describes the independent samples *t*-test between the control and autistic groups for all parameters.

**Figure 4:**
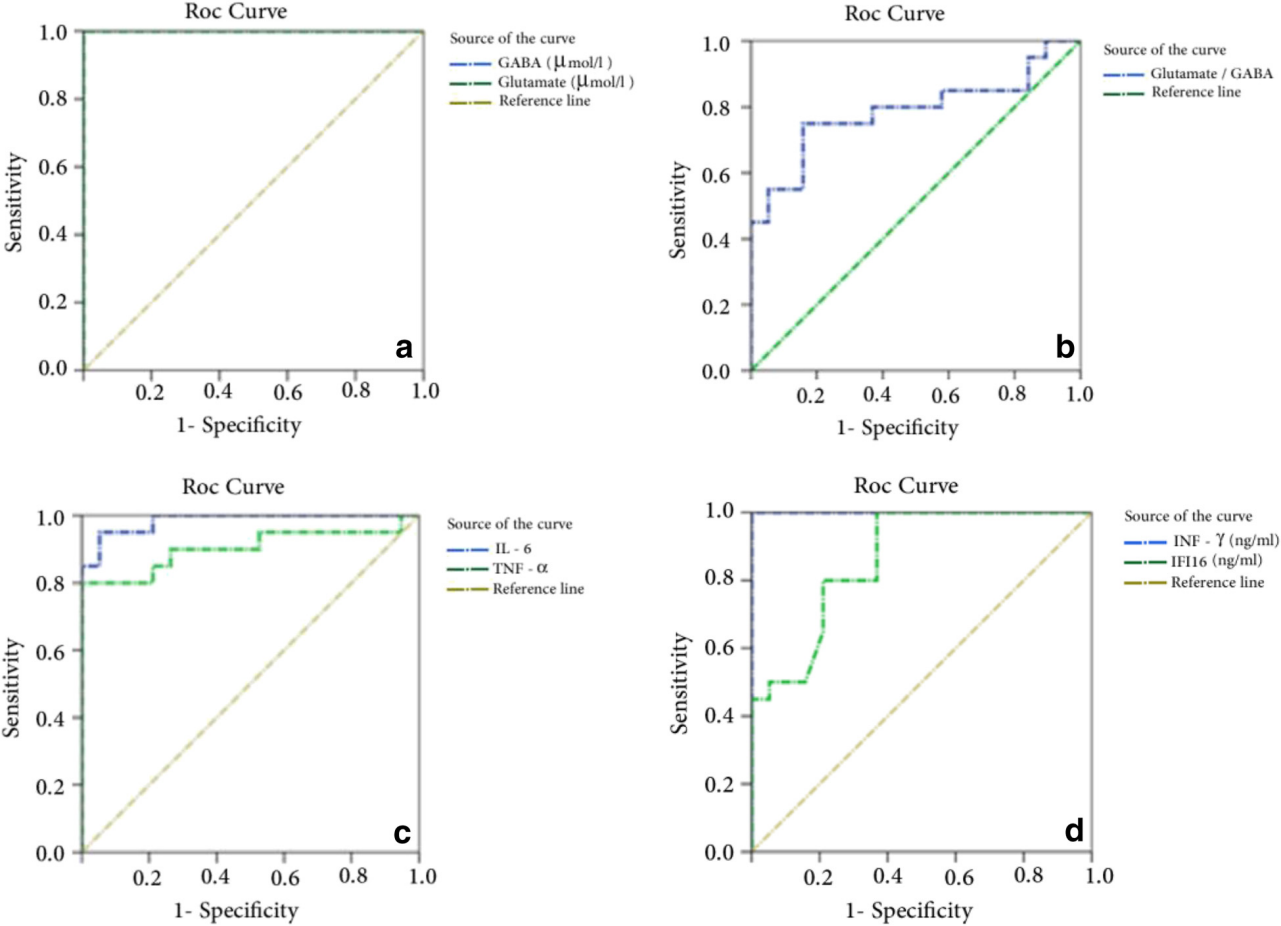
**Receiver operating characteristics curve (ROC) curve of: (a)** gamma aminobutyric acid (GABA; μmol/l) and glutamate (μmol/l), **(b)** glutamate/GABA ratio, **(c)** and **(d)** INF-γ (ng/ml) and IFI16 (ng/ml) in autistic groups.

Table 2 and Figure 4 (a-d) demonstrate the receiver operating characteristics (ROC) analysis data as area under the curve (AUC), cutoff values, specificity, and sensitivity of the measured parameters. All parameters exhibited AUC values close to 1 and satisfactory values of accuracy presented as high specificity and sensitivity.

**Table 2:** **Receiver operating characteristics (ROC) curve of all parameters in autistic groups**

	**Area under the curve**	**Best cutoff value**	**Sensitivity %**	**Specificity %**
GABA (μmol/l)	1.000	0.617	100.0%	100.0%
Glutamate (μmol/l)	1.000	131.110	100.0%	100.0%
Glutamate/GABA ratio	0.787	191.792	75.0%	84.2%
IL-6	0.984	333.840	95.0%	94.7%
TNF-α	0.903	285.530	80.0%	100.0%
INF-γ (ng/ml)	1.000	62.780	100.0%	100.0%
IFI16 (ng/ml)	0.864	1.860	100.0%	63.2%

Tables 3, 4 and 5 demonstrate the multiple regression analysis between the measured parameters using GABA, glutamate and glutamate/GABA as dependent variables respectively.

**Table 3:** **Multiple regression using stepwise method for gamma aminobutyric acid (GABA; μmol/l) as a dependent variable**

**Predictor variable**	**Beta**	***P*** **-value**	**Adjusted** ^**2**^ **R**	**Model**	
				**F-value**	***P*** **-value**
Glutamate (μmol/l)	0.617	0.001	0.973	687.641	0.001
Glutamate/GABA ratio	−0.589	0.001

**Table 4:** **Multiple regression using stepwise method for glutamate (μmol/l) as a dependent variable**

**Predictor variable**	**Beta**	***P*** **-value**	**Adjusted** ^**2**^ **R**	**Model**	
				**F-value**	***P*** **-value**
INF-γ (ng/ml)	0.594	0.001	0.869	85.107	0.001
TNF-α	−0.238	0.003			
IL-6	−0.221	0.026

**Table 5:** **Multiple regression using stepwise method for glutamate/gamma aminobutyric acid (GABA) ratio as a dependent variable**

**Predictor variable**	**Beta**	***P*** **-value**	**Adjusted** ^**2**^ **R**	**Model**	
				**F-value**	***P*** **-value**
GABA (μmol/l)	−1.545	0.001	0.935	183.521	0.001
Glutamate (μmol/l)	1.049	0.001			
IL-6	0.157	0.036			

## Discussion

GABA and glutamate are the main inhibitory and excitatory neurotransmitters in the human brain. Both have critical roles during early development of the nervous system, a stage when clinical presentation indicates that autism begins. Therefore, it is important to understand the functional status of GABAergic and glutamatergic neurotransmission in the plasma as the body fluid reflects brain physiology and pathology. Clarification of the relationship between abnormality in the absolute and relative GABAergic and glutamatergic synaptic activities and neuroinflammatory biomarkers represented by TNF-α, IL-6, IFN-γ, and IFI16 could help to find a target for pharmacological intervention.

Brain GABA and glutamate metabolism include a series of integrated and interconverted pathways. Glutamate, in addition to its importance in the clearance of brain ammonia through glutamine synthesis, serves as an important energy source through the tricarboxylic acid cycle (TCA) cycle after its deamination by glutamate dehydrogenase and oxidative decarboxylation by α-ketoglutarate dehydrogenase. Glutamate is converted to GABA by glutamic acid decarboxylase. Finally, GABA is metabolized to succinate by the combined reactions of GABA transaminase and succinic semialdehyde dehydrogenase. The significant increase of the absolute concentration of both neurotransmitters reported in the present study, together with the remarkable decrease in the glutamate/GABA ratio in autistic patients compared to healthy controls (Table 1), can be easily related to autistic characteristics. Since GABA derives from glutamate and glutamate derives from GABA, alterations in both neurotransmitters can affect each other. The unexpected elevation of GABA as an inhibitory neurotransmitter in autistic patients with a high rate of onset of epilepsy could be explained on the basis that high plasma GABA could be concomitant with lower brain GABA due to a smaller number or dysfunctional neuronal GABA receptors. GABA exerts its functions by binding to chloride-permeable ionotropic GABA_A_ receptors and metabotropic GABA_B_ receptors. This explanation can be acceptable because GABAergic transmission through receptors is achieved by different mechanisms such as modulation of GABA_A_ receptors and variation of intracellular chloride concentration together with alteration in GABA concentration [14]. Elevation of GABA level, which was recorded in the present study, can be easily related to the significant elevation of glutamate as substrate of glutamate decarboxylase. The suggested role of GABA receptors in the excitotoxicity or inhibitory/excitatory imbalance in autistic patient could be supported through the recent work of Han *et al*. [15] in which low doses of benzodiazepines increase inhibitory neurotransmission through positive allosteric modulation of post-synaptic GABA_A_ receptors, improved impaired social interaction, repetitive behavior, and cognitive ability in an animal model of autism. In contrast, negative allosteric modulation of GABA_A_ receptors impaired social behavior in wild-type mice, supporting the notion that reduced inhibitory neurotransmission may contribute to social and cognitive deficits as autistic characteristics.

The recorded decrease of IL-6 and TNF-α in plasma of autistic patients compared to control healthy participants (Table 1) could be a consequence of an early elevation of these cytokines in plasma followed by influx of both to the brain across the blood-brain barrier (BBB). This explanation can be supported by many studies that proved the elevation of IL-6 in autistic brains [16]. Although elevation of IL-6 is a repeated finding in autism, the exact mechanism by which an IL-6 increase may contribute to the pathogenesis of this disorder remains undefined. The association between IL-6 and low glutamate/GABA ratio (Table 5) observed in the present study, could help in clarifying this mechanism. Firstly, a key role for inhibitory/excitatory imbalance in autism is supported by the fact that 10 to 30% of autistic patients suffer from epilepsy [17]. This synaptic abnormality hypothesis was further supported by the identification of mutations affecting synaptic cell adhesion molecules, as well as synaptic proteins in autistic subjects [18-20]. Secondly, IL-6 elevation was found to stimulate excitatory synapse formation and impair the development of inhibitory synapses. This role of IL-6 was supported by the detection of a reduced post-excitatory inhibition in IL-6 overexpressed mice. Post-excitatory inhibition, which is usually measured by paired-pulse inhibition (PPI) has been suggested as being caused by a reduction in the release of glutamate from terminals of afferent axons [21,22]. This effect seems to be the result of an inhibition of calcium influx through presynaptic receptors, which play a critical role in the release of glutamate from synaptic vesicles on afferent stimulation [23,24]. Thirdly, mice with elevated IL-6 in the brain display many autistic features, including impaired cognitive abilities, deficits in learning, abnormal anxiety traits and habituations, as well as decreased social interactions [13].

The suggested increase of brain TNF-*α* and the association between this cytokine and the impaired glutamate/GABA ratio reported in the present study (Table 5) could be explained on the basis of the interesting relationship between neurons, glial cells and TNF-*α* leading to glutamate excitotoxicity [25]. In general, excitotoxicity is related to the activation of NMDA receptors by excessive glutamate. Brain cell death usually occurs after over stimulation of glutamate receptors due to impaired re-uptake of glutamate by the EAAT2/GLT-1 transporters on glial cells [26]. The expression of this transporter has been shown to be regulated by NF-*κ*B [27]. Pickering *et al*. [28] showed that increased levels of TNF-α might be easily related to glutamate excitotoxicity through the decrease of EAAT2/GLT-1 expression. TNF-α induces the classical I kappa B (I*κ*B) degradation pathway to trigger NF-*κ*B nuclear translocation and DNA binding to repress EAAT2 expression. In this situation, the presence of elevated TNF-α concentrations leads to elevated extracellular glutamate concentration, thereby increasing the risk of glutamate excitotoxicity. Elevated TNF-α concentrations could be also contributed to the decrease of the inhibitory transmission. Recently, an *in vitro* culture study in mature rat and mouse hippocampal neurons demonstrated that acute (45 minute) application of TNF-α induced a rapid and persistent decrease of inhibitory synaptic strength as well as a downregulation of cell-surface levels of GABA_A_ receptors [29].

Table 1 demonstrates the large significant increase of IFN-γ in plasma of autistic patients compared to healthy control participants. This increase was associated with the low glutamate/GABA ratio representing inhibitory/excitatory synaptic transmission (Table 5). This association could be reasonably accepted and explained on the basis of the key role of IFN-γ in the induction of neurotoxicity. Recently, it has been proposed that IFN-γ directly induces neuronal dysfunction through the formation of dendritic beads in mouse cortical neurons and the enhancement of glutamate neurotoxicity via AMPA but not NMDA receptors. IFN-γ forms a unique, neuron-specific, calcium-permeable receptor complex with AMPA receptor subunit GluR1. Through this receptor complex, IFN-γ phosphorylates GluR1 at serine 845 position by the JAK1-2/STAT1 pathway, increases Ca^+2^ influx and nitric oxide production, and subsequently decreases ATP production, leading to the dendritic bead formation. This explanation could provide a mechanisms through which inflammation could be related to glutamate excitotoxicity as an etiological signaling involved in autism pathology [30].

ROC analyses of glutamate, GABA, glutamate/GABA, TNF-α, IL-6, IFN-γ and IFI16 are presented in Table 2 and Figures 4 (a-d). All measured parameters demonstrated satisfactory sensitivity and very high specificity, which confirmed the hypothesis that autistic patients are characterized by excitotoxicity and suffer from neuroinflammation.

Multiple regression analysis, as one of the most powerful methods of data analysis, was used between the measured parameters to determine the relative contribution of impaired GABAergic and glutamatergic neurotransmission in the recorded neuroinflammation and to predict the role of the measured cytokines in the glutamatergic/GABAergic imbalance in autistic patients compared to healthy control subjects (Tables 3, 4 and 5).

The finding that the high plasma GABA levels are positively correlated to glutamate and glutamate/GABA levels (Table 3) led us to propose that the unexpected increase in GABA concentration in autistic patients is induced partly by glutamate bioavailability as substrate to glutamate decarboxylase (GAD) enzyme. This can be supported by the value of R^2^, which shows that 0.973 or 97% of the variance in GABA was related to the elevation of glutamate. Despite the elevated GABA in plasma of autistic patients, a dysfunction in inhibitory GABAergic transmission is suggested due to a reduced density of GABA_A_ receptors in the hippocampus [31,32], reduced GABA_A_ and GABA_B_ receptor protein subunits in the cerebellum, prefrontal Brodmann area 9 and parietal Brodmann area 40 [33,34], and a reduced expression of the GABA-synthesizing iso-enzymes GAD65 and GAD67 in the parietal and cerebellar cortices in autistic brains [35].

Table 4 demonstrates the stepwise multiple regression analysis using glutamate as dependent variable and TNF-α, IL-6, IFN-γ and IFI16 as independent variables. The value of R^2^ shows that 0.869 or almost 87% of the variance in glutamate was explained by, or related to, neuroinflammation as discussed above. While, IFN-γ cytokine was the most significantly related to glutamate excitotoxicity, IFI16 did not contribute.

## Conclusion

Based on the present study, it could be concluded that glutamate/GABA balance or excitatory/inhibitory balance is crucial for the functioning of the synapse. Multiple mechanisms may compromise this balance; neuroinflammation highly contributes to the imbalance and consequently to the etiology of autism.

## Abbreviations

GABA: gamma aminobutyric acid
TNF-α: tumor necrosis factor alpha
IL-6: interlukin-6
IFN-γ: interferon gamma
IFI16: interferon –induced protein 16
(mGlu R): metabotropic glutamate receptors
(iGlu R): ionotropic glutamate receptors
EDTA: ethylene-diamine-tetra-acetic acid
